# The Effect of Sports Rules Amendments on Exercise Intensity during Taekwondo-Specific Workouts

**DOI:** 10.3390/ijerph17186779

**Published:** 2020-09-17

**Authors:** Michał Janowski, Jacek Zieliński, Monika Ciekot-Sołtysiak, Agata Schneider, Krzysztof Kusy

**Affiliations:** 1 Department of Athletics, Strength and Conditioning, Poznan University of Physical Education, ul. Królowej Jadwigi 27/39, 61-871 Poznań, Poland; mjanowski@awf.poznan.pl (M.J.); jacekzielinski@wp.pl (J.Z.); ciekot@awf.poznan.pl (M.C.-S.); 2 Department of Cardiology Intensive Care Therapy and Internal Medicine, Poznan University of Medical Sciences, ul. Przybyszewskiego 49, 60-355 Poznań, Poland; aamille@wp.pl

**Keywords:** acceleration, blood lactate, breathing rate, energy expenditure, heart rate

## Abstract

We aimed to compare the change in exercise response to taekwondo-specific circuit workouts before and after competition rule amendments. A total of 240 workouts in 15 elite athletes were analyzed over two years. Physiological and kinematic data were gathered with the wireless Bioharness system along with capillary blood samples for lactate concentration. Progressive exercise tests until exhaustion were periodically performed to obtain reference data. The rule changes resulted in significant increases (mainly medium or large effects) in the physiological (2.9–14.4%) and kinematic (4.8–10.1%) response to taekwondo-specific workouts. The largest increases were for peak breathing rate (12.0%), energy expenditure (6.6%), blood lactate immediately after exercise (10.2%) and at the 30th min of recovery (14.4%), and peak kinematic activity (10.1%). Significant differences between taekwondo-specific workouts and tournament combats persisted after the shift from old to new rules, ranging from 2.4 to 38.5% for physiological and from 2.9 to 15.5% for kinematic variables. The largest workout–combat differences were revealed for post-exercise (15.9%) and recovery (38.5%) blood lactate, peak (−15.8%) and relative (−15.0%) breathing rate, and mechanical (13.5%) and physiological (14.2%) intensity. Our study suggests that the rule amendments significantly modify the exercise response to discipline-specific workouts and that taekwondo-specific training sessions do not fully recreate the tournament demands in terms of physiological and kinematic load.

## 1. Introduction

Sports rules are amended to strengthen the ethos of a sport discipline, adapt it to capabilities and needs of specific groups, attract spectators, respond to media pressure and interest, recruit athletes, or improve sports performance [1,2]. To achieve these goals, structural (space, time, equipment), functional (athletes’ behavior, obligations, rights, prohibitions, penalties), and other rules are being modified [1]. As shown in various sport disciplines, rule changes affect exercise response to competition tasks. In particular, the modifications impact exercise intensity or overall physical load, usually by increasing [3,4,5,6,7,8,9] or, less often, by decreasing it [10,11].

In recent years, World Taekwondo incorporated modern technology into the discipline, i.e., the Protector Scoring System (PSS), the instant Video Replay System (VRS), and relevant competition rule changes [12,13,14,15,16]. The latest revisions in Olympic taekwondo rules penalize non-fighting actions, the presence of “phantom striking”, and unnecessary leg elevation; limit possible fouls; and modify the scoring system. Each penalty, regardless of reason, results in a point gain for the opponent. Our previous research showed that these amendments of competition rules were linked to a noticeable shift in taekwondo combat profile toward greater body movement dynamics, higher intensity, and greater post-exercise fatigue than observed previously. Significant increments in kinematic variables (3–8%), heart rate (1.5–1.8%), energy expenditure (3–5%), overall physiological load (2–4%), and lactate concentration (15% immediately after combat and 25% in recovery) suggest that new rules are more demanding [5]. Apart from modifying the exercise response to taekwondo combat by increasing its intensity, the recent rule amendments have forced the coaches and athletes to modify their workout regime. Even if training sessions have not been changed in terms of exercise volume, repetitions, or exercise/rest ratio, the exercise intensity could have been altered due to subtle but crucial modifications in kicking mode.

According to earlier studies, taekwondo-specific workouts, and even simulated combats, although relatively intense, did not fully recreate the physiological response to tournament combats [17,18]. However, certain types of high-intensity interval training seem to be optimal for adapting to taekwondo competition [19,20,21,22]. Moreover, taekwondo-specific circuit training forms (i.e., incorporating the fighting techniques) are suggested to be preferred instead of non-specific exercise (e.g., running or cycling) to combine physical conditioning with fighting specificity [5]. Simulating tournament combat conditions during training sessions is still a very topical issue. The question arises as to how the rule changes have affected the exercise profile during taekwondo-specific workouts and how adequately the taekwondo-specific workouts reflect the demands of the tournament combat in this new situation. To the best of our knowledge, no scientific investigation has focused on the direct impact of sports rule amendments on the exercise response to any discipline-specific workouts.

This study aimed to compare the physiological and kinematic exercise response to taekwondo-specific circuit training sessions in highly trained taekwondo athletes before and after rule changes. We analyzed a large number of training sessions throughout a longer period (2 years) as opposed to previous studies [17,18,20,23,24,25,26,27,28,29,30,31,32,33,34,35]. We hypothesized that the rule amendments and related modifications in kicking modality would increase the exercise intensity of taekwondo-specific workouts and reduce the gap between real combat situation and workout in terms of physiological and kinematic response.

## 2. Materials and Methods

### 2.1. Participants

We monitored 15 highly trained taekwondo athletes aged 16–25 (19.9 ± 2.7) years, both male (*n* = 10) and female (*n* = 5), in a period of two years. They were black belt holders, members of the Polish national team, national champions, or medal winners in international tournaments. One athlete took part in the Olympic games, seven took part in continental championships (four medals), four took part in world championships (two medals), and nine took part in the Universiade (six medals). The study was performed according to the ethical standards laid down in the Declaration of Helsinki. The local bioethics committee at the Poznan University of Medical Sciences approved the study design (decision nos. 418/13 and 143/15). We informed the participants of the benefits and risks of the research procedures. All athletes or their parents (for participants aged < 18 years) provided signed, written consent before the commencement of the research project.

### 2.2. Experimental Design

Our main idea was to compare the exercise intensity during equivalent taekwondo-specific workouts conducted before and after taekwondo rule changes. The revisions of taekwondo rules were the result of an alteration planned beforehand by World Taekwondo (www.worldtaekwondo.org) (discussed among coaches and members worldwide). We performed the measurements in old rules from February 2016 until May 2017 and in new rules from June 2017 until March 2018. We monitored taekwondo-specific training sessions and tournaments using wearable bio-monitors to record physiological and kinematic parameters. Athletes underwent blood sampling directly before and after each training session or combat and after 30 min of recovery. Basic reference physiological indices were obtained on the basis of progressive cardiorespiratory treadmill tests until exhaustion performed between training subphases of the annual cycle. Identical procedures were used during both old and new rule periods. Importantly, the head coach and the members of the coaching staff remained the same during the whole study period. The coaches organized the taekwondo-specific training sessions in the same manner in old and new rules, i.e., they used the same kicking techniques, number of series/repetitions, and exercise-to-rest ratios. The only difference was kicking modality imposed by new rules. Therefore, potential confounding factors resulting from changing training approach/strategy, goals, methods, organizational solutions, etc. were minimized or avoided. Before the rule changes, athletes were allowed to keep the leg elevated to facilitate consecutive strikes or to deceive opponents. New rules prohibited such behavior. Consequently, coaches forbade athletes to execute “phantom striking” or continuous leg elevation during training sessions. Instead, athletes were ordered to set foot on the ground between strikes. Therefore, the strikes investigated in this study, that is, front kicks, turning kicks, and double roundhouse kicks, were first executed following the old (February 2016–May 2017) and then following the new (June 2017–March 2018) taekwondo rules.

The number of monitored training person sessions equaled 362 (195 under old rules and 167 under new rules), 122 of which were excluded. Finally, 240-person sessions (127 under old and 113 under new rules) were included in the analysis. We monitored each athlete for 8.4 ± 1.6 (range 6–11) sessions under old rules and 7.5 ± 1.0 (range 6–9) sessions under new rules. This difference was not significant (*p* = 0.075). Real combat monitoring included 16 tournaments, covering a total of 298 combats (142 under old and 156 under new rules). We excluded 51 combats and analyzed the remaining 247 (126 old vs. 121 new). Each athlete was monitored for 8.4 ± 3.2 (range 4–15) combats under old rules vs. 8.0 ± 3.4 (range 3–15) combats under new rules. This difference was also not significant (*p* = 0.783). Exclusion criteria for data recordings from training sessions and tournament combats were (i) insufficient exertion time (i.e., incomplete circuit, knock out, disqualification, injury, or any pause regardless of source longer than 5 min)—49 and 13 exclusions, respectively; (ii) prolonged time (above 5 min) of VRS challenge during tournament combat—0 and 18 exclusions; (iii) uncommon interferences (coach interventions, round shortening)—15 and 3 exclusions; (iv) technical issues (signal breaking, lag spikes, PSS or VRS malfunctions)—4 and 6 exclusions; and (v) insufficient time for blood sampling—54 and 11 exclusions. Laboratory measurements, aimed at obtaining reference cardiorespiratory responses to a standard progressive exercise, were performed six times under the old rules and three times under the new rules at intervals of approximately 3 months.

### 2.3. Laboratory Data Collection

Height and weight were measured using a digital stadiometer (Seca 285, Seca GmbH and Co., KG, Hamburg, Germany). Body fat and total lean body mass were measured with dual-energy X-ray absorptiometry (Lunar Prodigy device, Encore Software 16 Sp.1, GE Healthcare, Chicago, IL, USA). The same operator scanned the participants according to the standardized protocol described elsewhere [26]. Then, athletes performed an exercise treadmill tests until exhaustion. Briefly, after 3 min of standing on a treadmill (pulsar 3p, h/p/cosmos sports and medical GmbH, Nussdorf-Traunstein, Germany), the athletes started the exertion at 4 km/h, with an increase to 8 km·h^−1^ after 3 min. Afterward, the speed increased by 2 km·h^−1^ every 3 min. Athletes ran until volitional exhaustion was reached. Cardiorespiratory characteristics were measured breath-by-breath by an ergospirometer (MetaMax 3B-R2, Metasoft Studio Software 5.2.0, Cortex Biophysik, Leipzig, Germany), calibrated according to the manufacturer’s guidelines. We used the values of following cardiorespiratory variables as reference data for a training session and combat analysis: heart rate (HR; Polar Electro Oy, Kempele, Finland), breathing rate (BR), energy expenditure (EE), and maximum oxygen uptake ($\dot{V}$O_2max_). Maximal oxygen uptake was considered achieved if at least three of the following criteria were met: (i) a plateau in $\dot{V}$O_2_ despite an increase in minute ventilation, (ii) blood lactate concentration ≥ 9 mmol⋅L^−1^ in males and ≥7 mmol⋅L^−1^ in females, (iii) respiratory exchange ratio ≥ 1.10, (iv) heart rate ≥ 95% of the maximal value recorded in the previous tests, and (v) the Borg Scale rating ≥ 17. Athletes were verbally encouraged by coaches and the researchers conducting the test to achieve the highest possible running speed. Additionally, we determined exercise parameters at the ventilatory threshold (VT) and respiratory compensation point (RCP) on the basis of the ventilatory equivalents for oxygen ($\dot{V}$_E_/$\dot{V}$O_2_) and carbon dioxide ($\dot{V}$_E_/$\dot{V}$CO_2_), partial pressures of O_2_ (PETO_2_), and CO_2_ (PETCO_2_). Capillary blood samples were obtained from the fingertip before exercise, at exhaustion, and after 30 min of recovery to assay lactate concentration (LA) using the blood analyzer Biosen C-Line (EKF Diagnostics, Cardiff, United Kingdom).

### 2.4. Data Collection during Taekwondo-Specific Workouts and Tournaments

**Figure 1:**
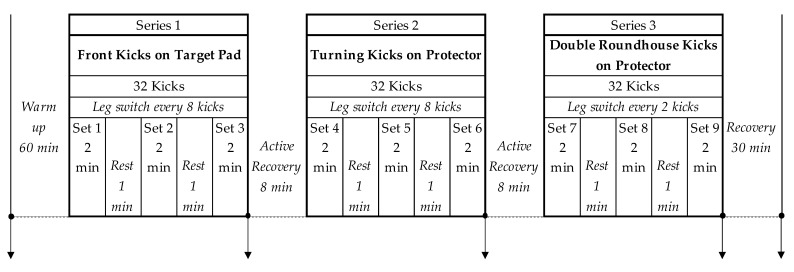
The structure of the analyzed taekwondo-specific circuits. Vertical downward arrows denote blood sampling.

Athletes wore bio-monitors (described below) through the whole training session or tournament day up to 30 min recovery period after the last workout exercise or combat, as described elsewhere [5]. The devices did not hamper their activity. The taekwondo-specific training sessions were organized in the form of a circuit containing nine exercise sets, each lasting 2 min (Figure 1). Typical combat consisted of three 2-min rounds with a 1-min rest period between each round.

Each taekwondo-specific circuit session was preceded by a standard ~40-min warm-up involving stretching (~15–20 min) and jogging (~15 min), followed by preliminary light to moderate kick exercises (~25 min). All circuits had the same structure and were divided into three series of exercise sets (“mini circuits”) separated by two 8-min active pauses. Each series, lasting 6 min net, was divided into three 2-min exercise sets separated by 1-min pauses to reflect the time structure of typical tournament combat. Each single exercise set contained taekwondo-specific kick techniques normally used during real combat—a minimum of 16 kicks per one leg, 32 kicks in total [27,28]. The names of the techniques are given in Figure 1. The circuit exercise was performed in pairs, where one athlete was an “attacker” (main exercise) and the second a “defender” (during the active 8-min pause). After each set, athletes changed their roles and exercise partners. The kicks were delivered on the chest protector or target pad held by the defending partner, depending on a particular technique. The athletes moved forward and backward during each exercise set to simulate real combat behavior. The total duration of each taekwondo-specific circuit was 40 min, including intervals between series and exercise sets, whereas net exercise time was 18 min.

Athletes used identical measuring equipment during training sessions and real combats to ensure the credibility of comparisons [17]. During tournaments and training sessions, we verified the ambient temperature with the K204 digital thermometer (Voltcraft, Wollerau, Switzerland). The temperatures ranged from 20.7 to 22.3 °C and from 19.9 to 22.6 °C, respectively. Capillary blood samples (fingertip) for lactate concentration were drawn five times during one training session: before the first exercise set, after each exercise set, and after the 30-min recovery period (Figure 1). During tournaments, blood was drawn before and immediately after each fight, and after the 30-min recovery period after the last combat. The physiological and kinematic indices were measured with a portable wireless piezoelectric recording system (Bioharness 3, Omnisense 3.9.7, Zephyr Technology Corp., Annapolis, MD, USA). According to the manufacturer’s definitions, the analyzed variables were as follows:

1. Peak activity (ACT_peak_)—squared root of (x^2^ + y^2^ + z^2^); x, y, and z are peak values of the three axial accelerations, where ~0.2 represents walking, ~0.5 jogging, ~0.8 running, and ~1.0+ sprinting.
2. Average activity (ACT_avg_)—squared root of (x^2^ + y^2^ + z^2^); x, y, and z are the averages of the three axial accelerations over the previous 1-s epoch, where ~0.2 represents walking, ~0.5 jogging, ~0.8 running, and ~1.0+ sprinting.
3. Physiological intensity (PHYS_int_)—range 0–10 (incremental step of 5%), where 0 is below 50% and 10 equals or exceeds 100% of maximum HR on the basis of the HR range of each athlete.
4. Physiological load (PHYS_load_)—the accumulation of the PHYS_int_ over time (average value multiplied by the total exertion time).
5. Mechanical intensity (MECH_int_)—displayed in the range of 0–10 (incremental step of 5%), where 0 denotes motionless, whereas 10 is equal to an acceleration of 3.0 g or greater.
6. Mechanical load (MECH_load_)—the accumulation of the MECH_int_ over time (average value multiplied by the total exertion time).
7. Training intensity (TRAIN_int_)—arithmetic average of PHYS_int_ and MECH_int_.
8. Training load (TRAIN_load_)—arithmetic average of PHYS_load_ and MECH_load_.
9. Energy expenditure—estimated according to the formula: EE (kcal) = Gender × (−55.0969 + 0.6309 HR + 0.1988 Weight + 0.2017 Age) + (1 − Gender) × (−20.4022 + 0.4472 HR − 0.1263 Weight + 0.074 Age). Gender—1 for male, 0 for female.

Other research teams [29,30,31] evaluated the reliability and validity of the Bioharness bio-monitor and recommended it as a high-precision tool in comparison with gold standards (*r* = 0.91, *p* < 0.01, CV (coefficient of variation) < 7.6% for heart rate and *r* = 0.94, *p* < 0.01 for acceleration).

### 2.5. Statistical Analysis

We compared the basic somatic and aerobic capacity profiles between different rule settings using the *t*-test for dependent samples. We cleaned raw training and combat data of any technical errors. The basic unit for analysis was one exercise set (average duration 120 ± 5 s) during training sessions or one combat round during tournaments (~120 s), as well as the last 10 min of the 30-min recovery following the last circuit or combat exertion. All values were presented as either mean ± standard deviation (general characteristics) or adjusted mean ± standard deviation of the mean (workout and combat data). We applied the analysis of covariance (ANCOVA) for repeated measures to identify the differences in circuit training and combat profiles between old and new competition rules. We adjusted the dependent variables for the following covariates that could potentially affect the magnitude of exercise response: (i) the order of exercise series or combat rounds (first, second, third), (ii) age category (junior, senior), and (iii) sex (male, female). Partial η^2^ was used as a measure of effect size and the following scale was adopted: small (0.01), medium (0.06), or large (0.14). An a priori calculation of required sample size revealed that at least 63 cases (workouts or combats) were needed to perform ANCOVA, assuming large effect size, *p*-level = 0.05, statistical power = 0.8, and three covariates, or 155 cases if medium effect size was assumed (G*Power 3.1.9.6, Franz Faul, Universität Kiel, Germany). The significance level was established at *p* < 0.05. We analyzed the data using Statistica 13.3 (TIBCO Software Inc., Palo Alto, CA, USA).

## 3. Results

Basic somatic and aerobic capacity profiles are displayed in Table 1. A vast majority of differences between old and new rule periods were nonsignificant. Exceptions were a decrease in oxygen uptake at the ventilatory threshold in males and increases in weight and absolute (but not percentage) lean body mass in the combined group.

**Figure 2:**
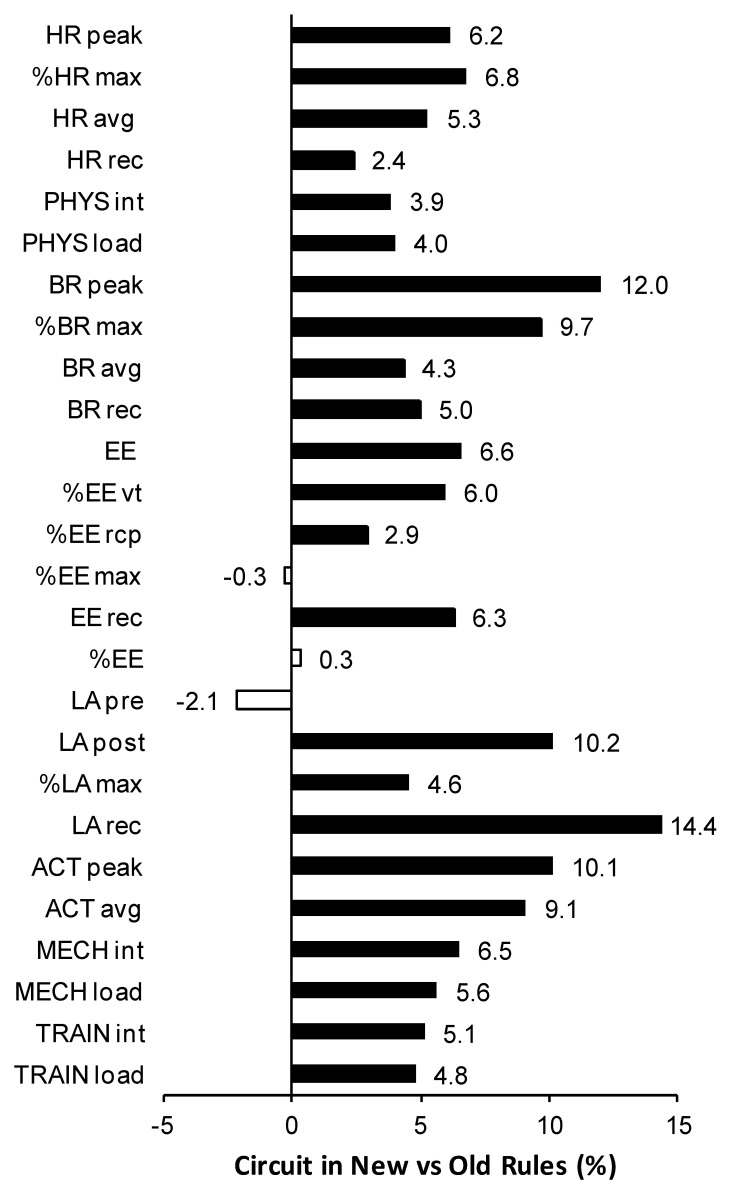
Percentage differences in exercise response to taekwondo-specific circuit workouts between new and old rule settings. Black bars denote statistically significant and white bars insignificant changes, as indicated in Table 2, Table 3, Table 4, Table 5 and Table 6. Positive numbers indicate higher values obtained in new vs. old rules. See table legends for the explanation of variable abbreviations.

**Figure 3:**
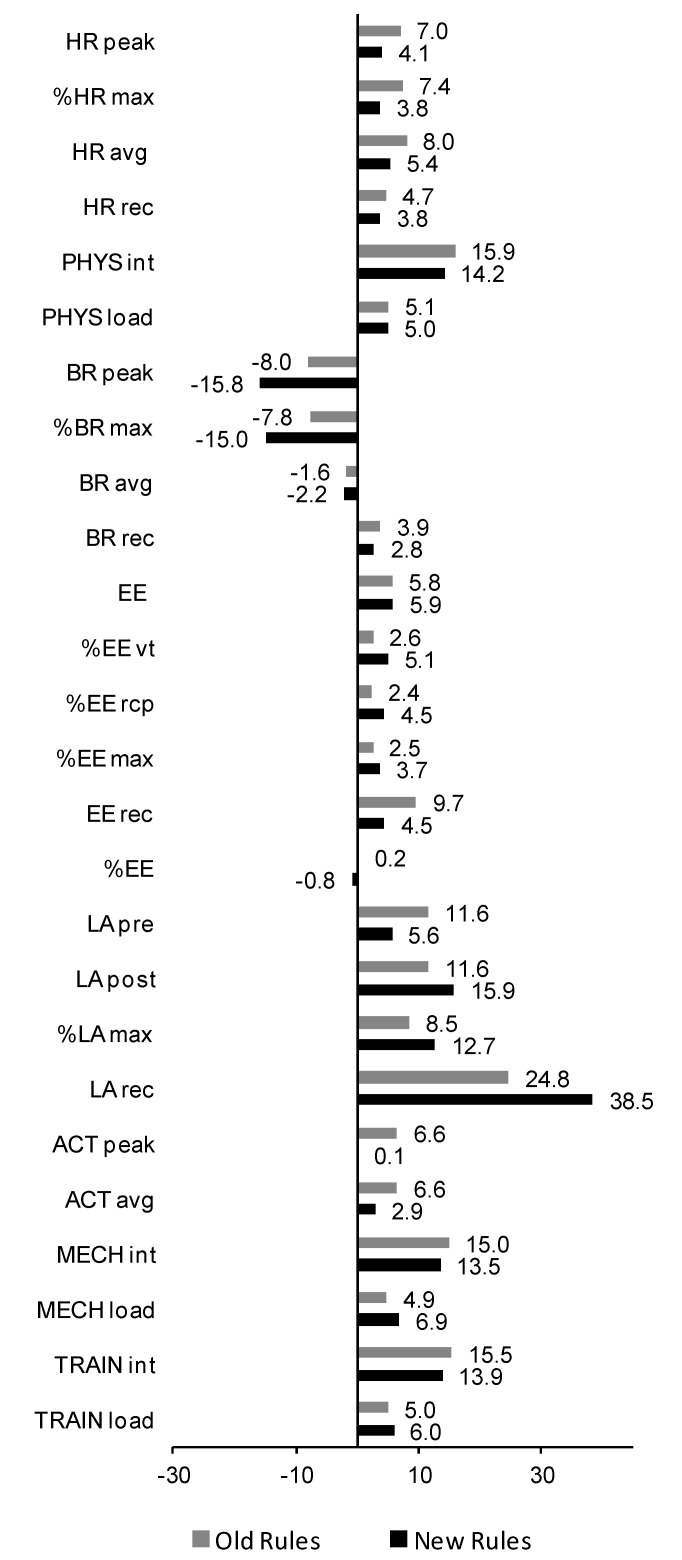
Percentage differences in exercise response between taekwondo-specific circuit sessions and real combat. Old rules are displayed as gray bars and new rules as black bars. Positive numbers indicate higher values obtained during combat vs. training sessions. See table legends for the explanation of variable abbreviations.

Peak, average, and recovery HR during circuit training were significantly higher under new rules compared with under old rules (Table 2), and the differences ranged between 2.4% and 6.8% (Figure 2). Under both old and new rules, peak, average, and recovery HR were significantly higher during tournament combat (~100% or >100% of maximum HR in the laboratory test until exhaustion) than taekwondo-specific training. The effect sizes, in general, were large, except for recovery HR (small). The same pattern of differences was revealed for physiological intensity (medium to large effect size) and load (small to medium effect size), with a ~4% difference between old and new training sessions (Figure 2) and ~4–16% difference between training sessions and real combats (Figure 3).

Peak and average breathing rate during circuit training were significantly higher (medium or large effect size) under new rules compared with under old rules (Table 3). The differences ranged between 4.3% and 12.0% (Figure 2). Peak and average breathing rate were significantly higher during circuit training when compared with real combat (medium or large effect size), with a smaller difference under old (~8%) compared with new (~15%) rules (Figure 3). The differences in breathing rate during post-exercise recovery were not significant or the effect size was small.

The estimated energy expenditure rate during the taekwondo-specific circuit (expressed as the percentage of expenditure at the ventilatory threshold and respiratory compensation point as well as absolute values) was significantly higher under new rules compared with under old rules overall (small to large effect size, 2.9–6.6% difference; Table 4 and Figure 2). The energy expenditure rate expressed as the percentage of expenditure at exhaustion during the progressive laboratory test was not different between rule settings. The average energy expenditure rate during the workouts was slightly above that for the respiratory compensation point ~104–107%). Absolute, but not percentage, energy expenditure during post-workout recovery was also significantly higher under new rules (medium effect size). In both old and new rule settings, the energy expenditure rate was in general higher during real combats than training circuits by ~0–6%, however, the effect size was small or even negligible (Table 4 and Figure 3).

Pre-exercise blood lactate concentration was not different between old and new rules (Table 5). Post-exercise values were significantly higher after training sessions administered under new rules compared with under old rules for both absolute and percentage values (4.6–10.4% difference; Figure 2), however, the effect size was small. Lactate levels during post-workout recovery were significantly higher under new rules compared with under old rule settings (14.4%, medium effect size). In both rule settings, lactate concentration was higher during tournaments than training sessions, with a small to medium effect for measurements directly after exercise and a large effect for recovery. The difference in post-exercise and recovery lactate concentration between training and combat increased from ~12% to 16% and from ~25% to ~39%, respectively, after rule amendments (Figure 3).

After the change from old to new rules, a significant increase by 4.8–10.1% was revealed in the levels of kinematic variables measured during taekwondo-specific circuits (Table 6 and Figure 2), especially in peak activity (medium to large effect size). Moreover, in old rules, the kinematic response to circuit training sessions was significantly weaker than the response to combat (small to large effect size). The differences (up to ~15%) persisted into new rules—circuit training was still less intensive than combat (medium and large effect size), except for peak activity (negligible difference).

## 4. Discussion

In this study, we found that the rule changes brought about visible increases in the physiological and kinematic exercise response to taekwondo-specific circuit training sessions. Moreover, significant differences in physiological and kinematic response to taekwondo-specific circuit sessions vs. real tournament combat persisted after the shift from old to new rules. Importantly, there was no change in the circuit protocol between both rule periods. However, one single but crucial qualitative modification, imposed by new rules, was introduced by coaches. Athletes were instructed to set foot on the ground between strikes, whereas under old rules athletes were allowed or even encouraged to remain in a fixed body position with an elevated leg before striking. The current taekwondo technique has become more dynamic and contains less isometric muscular activity such as keeping the leg elevated or holding the opponent. While the coaches in our study did not plan training sessions to become more intensive, they were forced to indirectly increase the circuit intensity after implementing new rule restrictions. In principle, this should be seen as a positive phenomenon because the taekwondo-specific circuit is a tool designed to prepare for exertion in real combat, which has become more intense since the rules were changed. However, there is also a negative side. Because the intensity has risen, the coaches should avoid a snowballing effect of cumulative training load, which has become substantially greater than in previous rule settings. It would be beneficial to take this into account when planning workouts and loads in a longer perspective. For example, coaches could schedule taekwondo-specific workouts closer to off-training days to prevent excessive fatigue and resulting overload, overtraining, or injuries.

In the past, multiple research teams investigated exercise response to taekwondo-specific training sessions [17,22,23,24,25,27,32,33,34,35,36,37,38] and high-intensity interval training [19,20,21,22] in male, female, cadet, junior, and elite adult taekwondo athletes. The training sessions required high-intensity exertion, resulting in blood lactate concentrations of 8.0–11.4 mmol·L^−1^, heart rate equal to 82–94% of the maximum, and a very high kinematic activity. Our results, including a large number of training sessions, are in general consistent with those studies that were only based on single or a few workouts. However, we obtained even higher lactate and heart rate values (Table 2), especially after the rule changes. Moreover, we characterized the exercise response with a much wider range of variables.

Our male athletes had a significantly lower oxygen uptake at the ventilatory threshold ($\dot{V}$O_2VT_) at the start of the new rules period. From the individual perspective of a particular athlete, the temporarily lower $\dot{V}$O_2VT_ means that with increasing exercise intensity, anaerobic metabolic processes (glycolysis) start to intensify earlier, including a raise in, for example, lactate levels, ventilatory equivalent for oxygen, and fatigue. Consequently, post-exercise recovery can be somewhat prolonged. However, during specific workouts and tournament combats, taekwondo athletes exclusively operate in the exercise intensity range between $\dot{V}$O_2RCP_ and $\dot{V}$O_2max_ [5]. These indicators of ability to high-intensity exercise were not different between both rules settings. We assume that the $\dot{V}$O_2VT_ was of much lesser significance for exercise response to taekwondo-specific tasks. It also seems that it was an incidental decline in $\dot{V}$O_2VT_, related to individual athletes, rather than a regular trend across multiple tests during the study period. In our previous report comparing combats in old and new rule settings [5], we did not reveal significant differences in $\dot{V}$O_2VT_, neither in male nor in female athletes. Moreover, in the combined group (Table 1), the differences in aerobic capacity between old and new rules were insignificant for all three $\dot{V}$O_2_ indicators. As we used data from the combined group in our analysis, only those characteristics were relevant as the background for workout or tournament exercise. Importantly, across the study period, we used each time reference dataset from the laboratory test closest to a particular training session or tournament; therefore, the workout parameters were adjusted to the current threshold or maximum aerobic parameters and any particular laboratory test was not decisive for the obtained results.

Some studies showed that exercise mode itself (technique or type of muscle contraction) affects physiological indices without modification of the external load. For example, in elite cross-country skiers, running, double poling on roller skis, and skating on roller skis resulted in significantly different heart rates and lactate concentrations, despite the same test protocol [39]. Moreover, equivalent dynamic and isometric muscular contractions in healthy males elicited different responses, i.e., rating of exertion, blood pressure, and rate pressure product were higher during isometric contraction, whereas breathing frequency, minute ventilation, oxygen uptake, and carbon dioxide output were higher during dynamic contraction [40]. In crawl swimming, it was revealed that the energy cost of swimming increased linearly along with changes in underwater torque (one of the biomechanical technique parameters) despite constant speed [41]. This supports the view that it is the change in kicking mode in taekwondo athletes that modified the exercise response to circuit training sessions in new competition rules, despite unchanged training session protocol.

There is very scarce research on the effect of rule changes on sport technique. Adam et al. pointed out that the rule changes in judo imposed important alterations in the efficiency of hand techniques, importance of leg techniques, as well as decline of all throwing techniques [42]. However, their study was focused on the combat strategy (choice/frequency of techniques during tournament combats), not on biomechanical analysis. Studies on the effect of fatigue or exercise intensity on sport technique are also interesting. Rule changes in taekwondo brought about increases in exercise intensity, and thus we assume that fatigue also increased. Aragones et al. found that among karate practitioners, noticeable kinematic changes emerged with fatigue development when repeating a complex action such as a karate front kick many times, despite the participants’ intention of performing identical repetitions [43]. Rusidiana et al. [44] revealed that fatigue affected the quality of a header’s motor performance in soccer. Some water sports-related studies [45,46,47] demonstrated that increased fatigue was related to technique including stroke characteristics and fingertip patterns. Prieske et al. concluded that fatigue was responsible for sex-specific knee motion strategies during jumping in elite volleyball players [48]. Grasaas et al. showed that high-intensity exhausting exercise resulted in less efficient technique in country skiers [49]. Kellis at al. revealed that fatigue induced significant impairment of soccer kick performance [50]. The above studies strongly indicate that an increase in intensity and resulting fatigue have a significant impact on movement technique including kinematic properties and the quality of motion. We suppose that such an effect also occurred in our taekwondo athletes.

With regards to the similarity between taekwondo-specific workout and tournament, the circuit training we analyzed did not fully recreate the combat situation in either rules version. An acceptable level of similarity between workout tasks and competition may be debatable. It may be also questioned as to whether a full identity of any training exercise with real combat is possible at all. Two studies are available on differences between real combat and taekwondo-specific training sessions, however, the authors reached opposed conclusions. Bridge et al. [17] demonstrated that taekwondo-specific exercise did not recreate the physiological responses of real combat (heart rate was higher by ~8% and post-exercise LA concentration higher by ~70% compared with during specific training). In contrast, Herrera-Valenzuela et al. [20] concluded that the training can successfully replicate the physiological demands of the tournament. They showed similar divergences in average heart rate (~2–8%) but much smaller differences in LA concentration (3–13%). In our study, we obtained divergences equal to ~5% in average heart rate and ~15% in lactate, thus closer to the latter research. The problem seems to be more complex than it appears and is not solely a matter of one or two physiological or kinematic variables. Similarity or discrepancy between training sessions and combat depends on a particular exercise protocol used, which may be focused either on technical/tactical skills or athletes’ specific conditioning [17,18,19,20]. Striking the balance between the two crucial components to find a “golden rule” is difficult. Shifting the burden to technical/tactical skills will result in lower workout intensity (inadequate to combat conditions), whereas focusing primarily on conditioning can result in technique deterioration. Additionally, other factors also play a role. For example, during training sessions, our athletes were forced to hit a moving opponent instead of a training kick-bag. We believe that the lack of a real opponent is the main constraint on combat imitation. During training sessions, athletes are usually not exposed to the risk of being hit upon by an opponent and, thus, are less emotionally involved. Bridge et al. [17]. observed a much higher increase in post-exercise blood adrenaline and noradrenaline levels during real combat than training sessions in international taekwondo athletes (4.5–4.8-fold difference), which suggests that the high-stress response present during official competition is hard, if not impossible, to fully recreate during simulated exercise. This may explain the differences in kinematic and physiological indices between combat and training sessions in our study, persisting despite the increase in circuit intensity after the rules change.

The discrepancies between training and combat may be unavoidable, however, they can be diminished by appropriate modification of quantitative and qualitative exercise parameters. Quantitative parameters, such as volume (exercise time, number of series/sets or repetitions), intensity (kicking frequency), and intervals between series/sets (short, long), are relatively easy to recreate during training. Qualitative parameters such as kicking techniques and their variations, non-fighting activities, opponent’s presence, and emotional involvement seem to be more challenging to imitate, but they could be crucial. In sports practice, it is important to design such a training protocol that, on the one hand, mimics the technical and tactical characteristics of real combat and, on the other hand, can replicate the kinematic and physiological response to combat. The appropriate balance between kinematic, physiological, technical, and tactical requirements ensures that taekwondo-specific exercise will be effective. An excessive emphasis on any of the above aspects may result in the loss of exercise specificity (similarity to real combat).

In our study, the breathing rate during training sessions significantly increased in new rules and was the only variable for which the discrepancy between training and combat deepened. In our previous study [5], athletes faced problems with low breathing frequency during combat. Unrestricted breathing is not possible during tournaments due to body dynamic movements, frequent explosive kicks, hand strikes, and blocks—all based on the Valsalva maneuver, i.e., the forceful attempt to exhale against a closed airway (holding the breath) to stabilize the trunk. Hence, breathing frequency is limited in combat as opposed to training where athletes can breathe relatively free. The breathing rate during circuit workouts increased after the rules change, whereas breathing during combat was limited. As a consequence, the “breathing gap” between the taekwondo-specific circuit and real combat widened. In new rules, we also observed a significant increase in workout variables measured after recovery, especially lactate levels, accompanied by a decrease in the discrepancies between training and combat. It seems that post-session fatigue persisted longer in the new than old rules period.

The results of our study can be seen from a broader perspective. In any sports discipline, the rule changes directly or indirectly affect specific training exercise load, not only the exertion during competition. Because the rule changes are an inevitable part of the development of virtually all sports, it would be beneficial to conduct similar research in other sports to reveal the discipline-specific direction and magnitude of effects. Coaches should be aware of rule changes and prepare their athletes for training intensity of a different magnitude than before. As revealed in this study, rule amendments have a direct impact on exercise response to discipline-specific workouts. Thorough recreation of real combat situations seems to be necessary to prepare athletes for the modified exercise load imposed by new rules. Recreating quantitative parameters (intensity, repetitions, etc.) is less cumbersome than qualitative factors (e.g., opponent, emotional stress). Coaches should be aware that differences in exercise response between competition and training sessions can be mitigated but will persist. Any changes in movement technique induced by rule amendments should be seriously taken into account when designing discipline-specific workouts.

Finally, the uniform nationality and club affiliation of the examined athletes may be seen as a limitation because one cannot be sure how other groups of taekwondo athletes have physiologically responded to the rule changes. Undoubtedly, local (national, club) factors could be moderators of the change in exercise response between the old and new rules. However, there are some arguments that our results can be generalized to a large extent. Having a homogenous group, we avoided the effect of some confounding factors. In a mixed group (various countries or sports clubs), it would be difficult to separate the “local” effects (specific coaching staff, different training methods/programs/schedules, social and physical environment, etc.) from the real effect of the rule changes. In our study, rule changes were “isolated”, and three potentially confounding factors were controlled statistically. Importantly, after the rule changes, the same structure of the taekwondo-specific circuit training was maintained, as well as the same coaching staff. This may be considered as a strength in the context of the study goal. Moreover, we examined individuals on a certain sports level who can be representative of the cohort of highly trained internationally experienced taekwondo athletes. The rule changes apply to taekwondo athletes worldwide, and thus they all have to adjust their technique and, most likely, they experience the same associated intensification of exertion in physiological and biomechanical terms.

## 5. Conclusions

After the competition rules change, the intensity of the taekwondo-specific circuit training sessions significantly increased due to modifications in the kicking technique itself without any change in exercise volume, repetitions, and exercise or rest duration. Moreover, the significant differences in exercise response between taekwondo-specific circuit training and real combat persisted or even deepened. Our study suggests that the amendments in sports regulations significantly modify the exercise response to specific training loads and that training sessions do not fully recreate the real combat situation.

## Figures and Tables

**Table 1:** Somatic and aerobic capacity profiles of the studied taekwondo athletes at the start of circuit training under old and new rules.

	Male		Female		Combined Group	
	Old Rules	New Rules	Old Rules	New Rules	Old Rules	New Rules
Age (years)	19.5 ± 3.3	20.5 ± 3.3	20.8 ± 1.5	21.8 ± 1.5	19.9 ± 2.8	20.9 ± 2,8
Experience (years)	8.6 ± 2.7	9.6 ± 2.7	9.2 ± 2.2	10.2 ± 2.2	8.8 ± 2.5	9.8 ± 2.5
Height (cm)	182.0 ± 5.9	182.0 ± 5.9	176.6 ± 8.1	176.6 ± 8.1	180.2 ± 6.9	180.1 ± 6.0
Weight (kg)	66.7 ± 11.1	70.1 ± 9.2	67.5 ± 12.5	68.9 ± 11.8	67.0 ± 11.1	70.1 ± 9.7 *
Lean body mass (kg)	53.8 ± 9.0	57.0 ± 7.6	44.6 ± 9.2	46.6 ± 7.4	50.7 ± 9.8	53.5 ± 8.9 **
Lean body mass (%)	80.7 ± 2.6	80.9 ± 2.2	66.4 ± 1.8	68.2 ± 2.0	76.0 ± 7.4	76.7 ± 6.5
Fat mass (kg)	9.8 ± 2.4	10.3 ± 2.3	19.5 ± 4.3	19.0 ± 4.2	13.0 ± 5.6	13.2 ± 5.2
Fat mass (%)	14.7 ± 2.6	14.5 ± 2.1	28.9 ± 2.7	27.7 ± 2.2	19.4 ± 7.4	18.9 ± 6.7
$\dot{V}$O_2VT_ (mL·min^−1^·kg^−1^)	41.0 ± 4.9	35.2 ± 3.7 **	30.0 ± 1.9	32.6 ± 5.7	37.3 ± 6.7	34.3 ± 4.4
$\dot{V}$O_2RCP_ (mL·min^−1^·kg^−1^)	47.9 ± 5.2	50.7 ± 3.3	39.0 ± 1.7	41.0 ± 3.2	44.9 ± 6.1	47.5 ± 5.7
$\dot{V}$O_2max_ (mL·min^−1^·kg^−1^)	54.1 ± 4.2	56.6 ± 4.1	44.6 ± 5.3	45.6 ± 4.8	50.9 ± 6.4	52.9 ± 6.8
LA_max_ (mmol·L^−1^)	8.4 ± 1.8	9.2 ± 1.7	8.0 ± 1.5	8.2 ± 1.3	8.3 ± 1.7	8.9 ± 1.7

Abbreviations: LA_max_—maximum blood lactate concentration after exercise test until exhaustion, $\dot{V}$O_2max_—maximum oxygen uptake, $\dot{V}$O_2RCP_—oxygen uptake at respiratory compensation point, $\dot{V}$O_2VT_—oxygen uptake at ventilatory threshold; *t*-test: * *p* < 0.01, ** *p* < 0.001—significantly different from old rules.

**Table 2:** Heart rate and relative physiological intensity/load during taekwondo-specific circuit training and combat—comparison between old and new rules.

	Circuit Old Rules	Circuit New Rules	Combat Old Rules	Combat New Rules	Circuit Old vs. New Rules		Circuit vs. Combat Old Rules		Circuit vs. Combat New Rules	
					*p*-Value	Effect Size	*p*-Value	Effect Size	*p*-Value	Effect Size
HR_peak_ (beats·min^−1^)	174.9 ± 7.1	185.7 ± 6.8	187.2 ± 11.5	193.3 ± 11.3	<0.001	0.37 (large)	<0.001	0.29 (large)	<0.001	0.14 (large)
%HR_max_	92.6 ± 4.3	98.9 ± 4.8	99.4 ± 5.8	102.6 ± 6.9	<0.001	0.32 (large)	<0.001	0.31 (large)	<0.001	0.09 (medium)
HR_avg_ (beats·min^−1^)	165.8 ± 6.7	174.6 ± 6.7	179.1 ± 12.3	184.0 ± 13.2	<0.001	0.30 (large)	<0.001	0.31 (large)	<0.001	0.17 (large)
HR_rec_ (beats·min^−1^)	102.8 ± 8.4	105.3 ± 8.1	107.6 ± 11.2	109.3 ± 10.7	0.022	0.02 (small)	0.002	0.05 (small)	<0.001	0.04 (small)
PHYS_int_ (au)	7.5 ± 0.5	7.8 ± 0.6	8.7 ± 0.6	8.9 ± 0.6	<0.001	0.06 (medium)	<0.001	0.51 (large)	<0.001	0.49 (large)
PHYS_load_ (au)	18.4 ± 2.0	19.2 ± 2.1	19.4 ± 2.1	20.1 ± 2.0	<0.001	0.03 (small)	<0.001	0.06 (medium)	<0.001	0.05 (small)

Abbreviations: HR_peak_—peak heart rate, %HR_max_—HR_peak_ as percentage of maximum heart rate obtained in progressive treadmill test, HR_avg_—average heart rate during training session or combat, HR_rec_—heart rate at the end of a 30 min recovery, PHYS_int_—physiological intensity, PHYS_load_—physiological load.

**Table 3:** Breathing rate during taekwondo-specific circuit training and combat—comparison between old and new rules.

	Circuit Old Rules	Circuit New Rules	Combat Old Rules	Combat New Rules	Circuit Old vs. New Rules		Circuit vs. Combat Old Rules		Circuit vs. Combat New Rules	
					*p*-Value	Effect Size	*p*-Value	Effect Size	*p*-Value	Effect Size
BR_peak_ (breaths·min^−1^)	43.7 ± 3.1	48.9 ± 2.9	40.2 ± 4.6	41.2 ± 4.6	<0.001	0.42 (large)	<0.001	0.16 (large)	<0.001	0.50 (large)
%BR_max_	69.7 ± 8.8	76.4 ± 10.0	64.3 ± 10.0	65.0 ± 9.8	<0.001	0.11 (medium)	<0.001	0.08 (medium)	<0.001	0.25 (large)
BR_avg_ (breaths·min^−1^)	36.5 ± 2.7	38.1 ± 2.6	35.9 ± 5.0	37.3 ± 4.8	<0.001	0.08 (medium)	0.040	0.01 (small)	0.004	0.02 (small)
BR_rec_ (breaths·min^−1^)	16.5 ± 2.2	17.3 ± 2.2	17.1 ± 3.1	17.8 ± 2.6	0.003	0.03 (small)	0.130	<0.01 (negligible)	0.266	<0.01 (negligible)

Abbreviations: BR_peak_—peak breathing rate, %BR_max_—BR_peak_ as percentage of maximum breathing rate obtained in progressive treadmill test, BR_avg_—average breathing rate during training session or combat, BR_rec_—breathing rate at the end of a 30 min recovery.

**Table 4:** Energy expenditure during taekwondo-specific circuit training and combat—comparison between old and new rules.

	Circuit Old Rules	Circuit New Rules	Combat Old Rules	Combat New Rules	Circuit Old vs. New Rules		Circuit vs. Combat Old Rules		Circuit vs. Combat New Rules	
					*p*-Value	Effect Size	*p*-Value	Effect Size	*p*-Value	Effect Size
EE_avg_ (kcal·kg^−1^·h^−1^)	14.1 ± 1.2	15.0 ± 1.1	13.2 ± 2.5	15.9 ± 2.9	<0.001	0.14 (large)	<0.001	0.03 (small)	<0.001	0.04 (small)
%EE_vt_	132.4 ± 22.6	140.5 ± 18.0	135.8 ± 26.5	147.4 ± 23.0	<0.001	0.04 (medium)	0.06	<0.01 (negligible)	<0.001	0.03 (small)
%EE_RCP_	104.2 ± 11.9	107.2 ± 9.6	106.8 ± 20.4	112.1 ± 15.5	0.010	0.02 (small)	0.04	0.01 (small)	<0.001	0.03 (small)
%EE_max_	91.8 ± 11.7	91.5 ± 7.6	94.1 ± 17.6	94.9 ± 13.2	0.720	<0.01 (negligible)	0.03	0.01 (small)	<0.001	0.02 (small)
eE_rec_ (kcal·kg^−1^·h^−1^)	5.3 ± 0.6	5.6 ± 0.6	5.8 ± 1.4	5.8 ± 1.2	<0.001	0.08 (medium)	<0.001	0.06 (medium)	0.094	<0.01 (negligible)
%ee_rec_	37.2 ± 4.4	37.2 ± 4.3	37.3 ± 10.7	36. 9 ± 9.7	0.984	<0.01 (negligible)	0.944	<0.01 (negligible)	0.795	<0.01 (negligible)

Abbreviations: EE_avg_—average energy expenditure rate, %EE_VT_—percentage of energy expenditure rate measured at the ventilatory threshold, %EE_RCP_—percentage of energy expenditure rate measured at respiratory compensation point, %EE_max_—percentage of energy expenditure rate measured at exhaustion in the progressive treadmill test, EE_rec_—absolute energy expenditure rate at the end of a 30 min recovery after the last series of the circuit or tournament combat, %EE_rec_—EE_rec_ as percentage of EE_avg_.

**Table 5:** Lactate concentration during taekwondo-specific circuit training and combat—comparison between old and new rules.

	Circuit Old Rules	Circuit New Rules	Combat Old Rules	Combat New Rules	Circuit Old vs. New Rules		Circuit vs. Combat Old Rules		Circuit vs. Combat New Rules	
					*p*-Value	Effect Size	*p*-Value	Effect Size	*p*-Value	Effect Size
LA_pre_ (mmol·l^−1^)	2.0 ± 0.5	2.0 ± 0.4	2.2 ± 0.9	2.1 ± 1.0	0.451	<0.01 (negligible)	0.01	0.03 (small)	0.261	0.01 (small)
LA_post_ (mmol·l^−1^)	10.0 ± 2.8	11.1 ± 2.9	11.2 ± 2.4	12.8 ± 2.3	<0.001	0.03 (small)	<0.001	0.03 (small)	<0.001	0.07 (medium)
%LA_max_	120.8 ± 36.8	126.3 ± 34.5	131.1 ± 29.1	142.4 ± 27.6	0.039	0.01 (small)	0.004	0.02 (small)	<0.001	0.06 (medium)
la_rec_ (mmol·l^−1^)	2.2 ± 0.5	2.5 ± 0.44	2.7 ± 0.8	3.5 ± 1.0	<0.001	0.11 (medium)	<0.001	0.15 (large)	<0.001	0.32 (large)

Abbreviations: LA_pre_—resting blood lactate concentration, LA_post_—post-exercise blood lactate concentration, %LA_max_—post-exercise lactate concentration as a percentage of maximum concentration at exhaustion in progressive treadmill test, LA_rec_—blood lactate concentration at the end of a 30 min recovery.

**Table 6:** Kinematic response to taekwondo training and combat—comparison between old and new rules.

	Circuit Old Rules	Circuit New Rules	Combat Old Rules	CombatNew Rules	Circuit Old vs. New Rules		Circuit vs. Combat Old Rules		Circuit vs. Combat New Rules	
					*p*-Value	Effect Size	*p*-Value	Effect Size	*p*-Value	Effect Size
ACT_peak_ (m·s^−2^)	12.3 ± 1.5	13.6 ± 1.7	13.2 ± 2.5	13.6 ± 2.4	<0.001	0.14 (large)	<0.001	0.04 (small)	0.944	<0.01 (negligible)
ACT_avg_ (m·s^−2^)	7.1 ± 0.5	7.7 ± 0.5	7.5 ± 0.8	8.0 ± 0.6	<0.001	0.28 (large)	<0.001	0.11 (medium)	<0.001	0.04 (small)
MECH_int_ (au)	7.1 ± 0.5	7.5 ± 0.6	8.1 ± 0.7	8.5 ± 0.6	<0.001	0.17 (large)	<0.001	0.43 (large)	<0.001	0.46 (large)
MECH_load_ (au)	16.8 ± 1.8	17.8 ± 1.8	17.6 ± 2.2	19.0 ± 1.9	<0.001	0.07 (medium)	<0.001	0.04 (small)	<0.001	0.10 (medium)
TRAIN_int_ (∑au)	7.3 ± 0.5	7.6 ± 0.5	8.4 ± 0.6	8.7 ± 0.5	<0.001	0.13 (medium)	<0.001	0.53 (large)	<0.001	0.52 (large)
TRAIN_load_ (∑au)	17.6 ± 1.7	18.5 ± 1.8	18.5 ± 2.0	19.6 ± 1.8	<0.001	0.06 (medium)	<0.001	0.06 (medium)	<0.001	0.09 (medium)

Abbreviations: ACT_peak_—peak mechanical activity, ACT_avg_—average mechanical activity, MECH_int_—mechanical intensity, MECH_load_—overall mechanical load, TRAIN_int_—average summary of exertion intensity, TRAIN_load_—average summary of exertion loads, au – arbitrary units.
